# Andrographolide in atherosclerosis: integrating network pharmacology and *in vitro* pharmacological evaluation

**DOI:** 10.1042/BSR20212812

**Published:** 2022-07-01

**Authors:** Shuai Shi, Xinyu Ji, Jingjing Shi, Shuqing Shi, Fei She, Qiuyan Zhang, Yu Dong, Hanming Cui, Yuanhui Hu

**Affiliations:** 1 Guang’anmen Hospital, China Academy of Chinese Medical Sciences, Beijing, People’s Republic of China; 2 Institute of Basic Research in Clinical Medicine, China Academy of Chinese Medical Sciences, Beijing, People’s Republic of China; 3 Department of Cardiology, Beijing Tsinghua Changgung Hospital, School of Clinical Medicine, Tsinghua University, Beijing, China

**Keywords:** Andrographolide, Atherosclerosis, Network pharmacology, Reverse cholesterol transport

## Abstract

Objective: *Andrographis paniculata (Burm.f.) Nees* is a medicinal plant that has been traditionally used as an anti-inflammatory and antibacterial remedy for several conditions. Andrographolide (AG), the active constituent of *A. paniculata (Burm.f.) Nees*, has anti-lipidic and anti-inflammatory properties as well as cardiovascular protective effects. The present study aimed to explore the effects of AG on the progression of atherosclerosis and to investigate related mechanisms via network pharmacology.

Materials and methods: Compound-related information was obtained from the PubChem database. Potential target genes were identified using STITCH, SwissTargetPrediction, Bioinformatics Analysis Tool for Molecular mechANism of Traditional Chinese Medicine, and Comparative Toxicogenomics Database. Genes involved in atherosclerosis were obtained from DisGeNet and compared with AG target genes to obtain an overlapping set. Protein–protein interactions were determined by STRING. Gene ontology (GO) analysis was performed at WebGestalt, and Kyoto Encyclopedia of Genes and Genomes (KEGG) pathway enrichment was analyzed using Metascape. The final network showing the relationship between compounds, targets, and pathways was constructed using Cytoscape. After that, oxLDL-induced RAW264.7 cells were used to further validate a part of the network pharmacology results.

Result: Eighty-one potential AG target genes were identified. PPI, GO, and KEGG enrichment revealed genes closely related to tumor progression, lipid transport, inflammation, and related pathways. AG improves the reverse cholesterol transport (RCT) through NF-κB/CEBPB/PPARG signaling in oxLDL-induced RAW264.7 cells.

Conclusion: We successfully predict AG’s potential targets and pathways in atherosclerosis and illustrate the mechanism of action. AG may regulate NF-κB/CEBPB/PPARG signaling to alleviate atherosclerosis.

## Introduction

Reverse cholesterol transport (RCT) is one of the important mechanisms associated with atherosclerosis (AS) and various cardiovascular diseases. Lipid infiltration is a key risk factor for the increased incidence of atherosclerosis, which leads to stroke, coronary artery disease (CAD), and carotid intima-media thickness [1]. Ischemic stroke and CAD arising from atherosclerosis is the leading cause of death and morbidity worldwide [2]. The annual incidence of sudden cardiac death is 69/100,000 in the United States, and the number of new stroke cases is over 2 million per year in China [3,4]. The current guidelines for treating AS prescribe statins, such as atorvastatin, simvastatin, and lovastatin, as the primary interventions for atherosclerotic cardiovascular disease (ASCVD). However, musculoskeletal weakness is one of the commonly reported side effects during the treatment [5]. For some ASCVDs induced by autoimmune inflammatory diseases like systemic lupus erythematous, evidence of the beneficial effects of statin therapy is limited [6]. Moreover, regular use of aspirin for cardiovascular disease primary prevention is associated with the risk of severe bleeding [7]. Therefore, the design and development of new drugs targeting ASCVD remain an active aspect of biomedical research.

**Figure 1:**
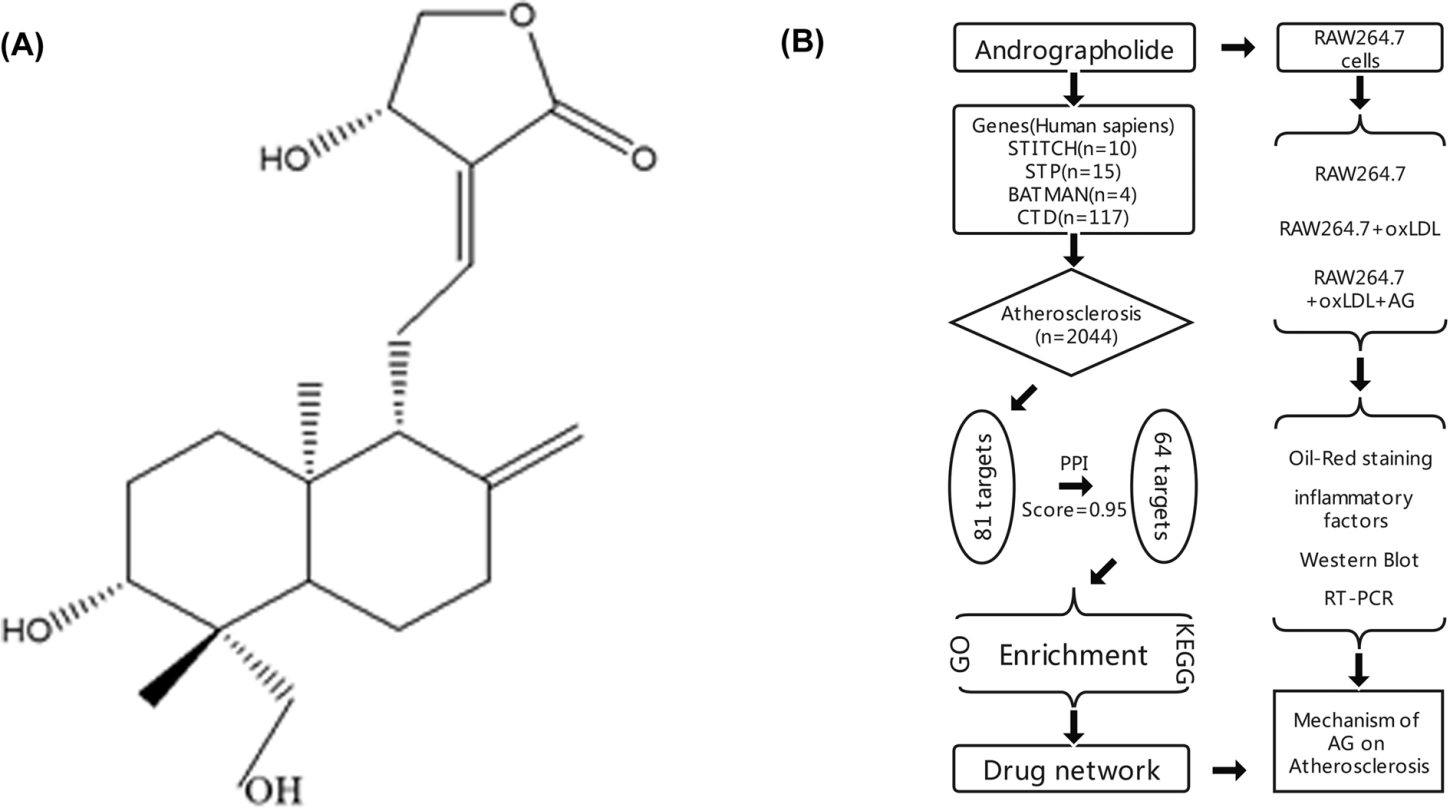
Structure of AG and flow chart of the experiment (**A**) Structure of AG downloaded from PubChem database (https://pubchem.ncbi.nlm.nih.gov; PubChem CID: 5318517); (**B**) flow chart of the process.

One of the successful strategies for novel drug discovery is identifying active compounds from medicinal plants. *Andrographis paniculata* is a medicinal herb from Acanthaceae used in traditional Chinese medicine (TCM) with thousands of years of history. Andrographolide (G), an active ingredient with diterpene lactone structure in *A. paniculata* (Figure 1A), has been demonstrated to have anti-inflammatory properties in *in vitro* and *in vivo* studies [8–11]. AG and its derivatives effectively suppress the development of inflammation-related diseases like stroke, rheumatoid arthritis, and joint pain by inhibiting transcription factor NF-κB signaling pathways [12,13]. Meanwhile, other potential therapeutic applications of andrographolide (AG) and its derivatives, such as antiviral function, antioxidative stress, antitumor, organ protection, and antihyperglycemia, have also been reported [14–17]. Recent evidence indicates that the antiatherosclerosis properties of AG are related to its inhibition of lipogenic gene expression, adipocyte formation, and macrophage foam cell formation [18,19]. Taken together, these findings suggest that the functions of AG are multi-targeted, and network analysis will be necessary for further understanding the mechanisms behind its activities.

The economic and time costs of drug development are large. Network pharmacology is a new method for systemically analyzing and predicting the mechanisms of an active compound or a drug. It was established by updating the research paradigm from the current ‘one target, one drug’ mode to the new ‘network target, multi-components’ mode [20]. The potential candidate targets of an active compound are first selected from different databases, followed by incorporation of genetic information obtained from the disease database. Next, based on the enrichment analysis results of genes, a functional network is generated to facilitate mechanistic studies or new drug development. This method greatly helps decrease the drug attrition rate, and thus, it is vital for rational and cost-effective drug development [21]. It is also suitable for exploring existing mechanisms for developing new drugs. In a similar manner, we have tried to systematically evaluate the mechanism and properties of AG in atherosclerosis by data mining.

In the present study, we first retrieve the target genes corresponding to AG (the structure shown in Figure 1A) through the database. Second, we select genes duplicated in the disease database and conduct related analyses and enrichment. Third, we build the above results into a network. Finally, an oxidized low-density lipoprotein (oxLDL)-induced RAW264.7 model was constructed for further validation. The specific process is shown in Figure 1B.

## Methods

### Plant extract

AG was extracted from *A. paniculata* (Burm.f.) Nees as a high purity crystalline powder (purity: 98.4%, LOT. 190402) by the Sichuan Wenlong Pharmaceutical Co., Ltd (Sichuan, China), with inspection Report No. C-01-19013.

### Chemical, physical, and toxic properties of AG

The chemical and physical properties related to AG structure and toxicological properties were obtained from the PubChem database (https://pubchem.ncbi.nlm.nih.gov). PubChem is an open chemistry database mainly for small molecules, but larger molecules also are included. The records of PubChem are accumulated from hundreds of data sources, such as chemical vendors, journal publishers, and government agencies [22].

Acute toxicology tests in mice were carried out following the protocol of Liao et al. We divided male *Apoe-/-* mice into three random groups, each containing eight mice. Two different doses of AG (1000 and 5000 mg/kg) were administered to two different groups of mice. Animals were provided with free access to food and water. Throughout the 14-day experiment, the mice were monitored for any mortality or behavioral changes. The behavioral changes observed included hyperactivity, tremors, ataxia, convulsions, salivation, diarrhoea, lethargy, sleep, and coma. The study was performed in compliance with the Animal Experimental Ethics Committee of Guang'anmen Hospital, China Academy of Chinese Medical Sciences.

### Target acquisition and screening

Different types of targets matched to AG were collected from the following databases, which are often used in the literature for monomer research. (1) STITCH (http://stitch.embl.de/cgi/input.pl?UserId=BzUtaFPWtqdq&sessionId=l7vk7fHggCm6) enables users to view the binding affinities of a chemical in an interaction network and the potential effects of the chemical on its interaction targets [23]; (2) Swiss Target Prediction (STP, http://old.swisstargetprediction.ch/) measures the similarity between new molecules and known ligands to predict their targets accurately [24]; (3) Bioinformatics Analysis Tool for Molecular mechANism of Traditional Chinese Medicine (BATMAN-TCM, http://bionet.ncpsb.org/batman-tcm/) is an online Bioinformatics Analysis Tool for TCM ingredients’ target prediction and functional analysis of targets, including biological pathways and enrichment analyses and visualization [25]; (4) Comparative Toxicogenomics Database (CTD, http://ctdbase.org/) [26] is a premier public resource with manually curated associations between chemicals, targets, diseases, and so on based on literature.

After collection, we input the gene symbols to Uniprot (https://www.uniprot.org/) for gene names and Uniprot IDs. Uniprot ID is an important identification when performing protein alignment between different databases. Genes related to atherosclerosis were obtained from the following databases: (1) DisGeNet (http://www.disgenet.org/search), a versatile platform for various research purposes like investigating the molecular underpinnings of specific human diseases and their comorbidities and analysing the properties of disease genes, etc. [27]; (2) DrugBank database (https://www.drugbank.ca/), a robust and comprehensive bioinformatics database containing data on drugs and disease [28]; (3) Online Mendelian Inheritance in Man (OMIM) database (https://www.omim.org/), an authoritative, timely, and comprehensive database about human genes and genetic diseases [29]; (4) Therapeutic Target Database (http://db.idrblab.net/ttd/), which provides data on therapeutic protein and nucleic acid targets for the targeted disease [30]. *Homo sapiens* was selected as the species. After targets from databases were compared with targets matched to AG, the final81 targets were successfully identified.

### Protein–protein interaction (PPI)

STRING (https://string-db.org/) is a protein-related database that collects, scores, and integrates many publicly available sources of protein–protein interaction information and complements information with computational predictions. Finally, a global network of PPI, including direct (physical) and indirect (functional) interactions, is generated [31].

*Homo sapiens* was selected as the organism after entering the above target list into the multiple-protein search. To decrease the complexity of the network, 0.95 was set as the minimum required interaction score, and disconnected nodes in the network were hidden. Network parameters were adjusted to get a clearer structure in Cytoscape (v3.6.0; https://cytoscape.org/) [32].

### Gene ontology (GO) analysis

The gene ontology (GO) project is a result of efforts to align the functional description of gene products in various databases. GO has developed a standard language (ontologies) with a tertiary structure, which is defined according to the gene product's relevant molecular functions, biological pathways, and cytological components. WEB-based GEne SeT AnaLysis Toolkit (WebGestalt, http://www.webgestalt.org/option.php) supports 324 gene identifiers from various platforms and computationally analyses 150937 functional categories from public databases [33]. After choosing overrepresentation enrichment analysis (OSA), UniProt IDs of 81 genes were uploaded, and ‘genome’ was selected as the reference set.

### Kyoto Encyclopedia of Genes and Genomes (KEGG) pathway enrichment

Metascape (http://metascape.org/gp/index.html#/main/step1) is a web-based platform designed to provide a comprehensive gene list annotation and pathway analysis resource for researchers [34]. During the operation, we selected the customized analysis for the enrichment of 81 target genes in KEGG. KEGG, including the GENES, PATHWAY, and LIGAND databases, is a knowledge base for the systematic analysis of gene functions; it links genomic information with different levels of functional information.

### Compound-target-pathway network construction

“Compound-Target-Pathway network” was established by Cytoscape 3.6.0 software based on the results in the Metascape platform. The characteristics of multiple targets and multiple pathways of AG were displayed and analysed through the entire complex compound, target, and disease network.

### Cell culture and treatment

RAW264.7 cells were acquired from the Cell Resource Center, Peking Union Medical College (the headquarters of National Infrastructure of Cell Line Resource, NSTI) on March 15, 2018. The cell line was routinely tested for the absence of mycoplasma contamination by PCR and culture. Its species origin was authenticated using PCR. The identity of the cell line was confirmed with STR DNA profiling (FBI, CODIS). All the results are available online at the website (http://cellresource.cn). RAW264.7 cells were maintained in a 25-cm^2^ cell culture flask with a 2 μm vent cap at 37°C in a CO_2_ incubator (5% CO_2_, 95% air). Cells were cultured in 8 ml DMEM (Cat No. M1805, CELL technologies, China) supplemented with 10% Defined Fetal Bovine Serum (FBS, Cat No. SH30070.03, Hyclone laboratories., lnc, U.S.A.), 1.25% L-glutamine, and 2% penicillin/streptomycin. RAW 264.7 macrophages were seeded in 6-well plates (2 × 10^5^/well) in 10% FBS DMEM as described [32,33]. The oxLDL was purchased from Shanghai yuanye Bio-Technology Co., Ltd (Cat No. S24879-2mg, China). Cells were classified into three groups: untreated RAW264.7 cells (RAW264.7), oxLDL treated foam cells (RAW264.7+oxLDL), and oxLDL+ 50 μmol/L AG treated foam cells (RAW264.7+oxLDL+AG). After growing to 80% confluency, cells were stimulated with 50 μg/ml oxLDL in a serum-free medium for 24 h. In parallel, 50 μmol/L AG was added together with oxLDL for 24 h. The final concentration of oxLDL was decided by preliminary experiments and previous research [35–37].

### Oil-red staining

For lipid staining, Oil Red O powder (Cat No. O0625-100G, SIGMA-ALDRICH, U.S.A.) was used according to the BioVision manual. After the indicated treatments, cells were fixed with 4% paraformaldehyde (PFA, Ref No. BL539A, biosharp, Beijing Labgic Technology Co., Ltd, China) in the indicated groups for 30 min and stained with Oil Red O solution for 15 min at 25 ± 2°C. Finally, The cells were observed with a microscope (Olympus IX70) equipped with a camera at 200× magnification. The percentage of Oil Red O positive area was measured using the ImagePro Plus software.

### Determination of inflammatory cytokines in cell supernatant

Cell supernatant was collected following each treatment. Meanwhile, the Bio-Plex System was warmed up for at least 30 min. A single vial of standards was reconstituted in 500 μl of a diluent similar to the final sample type or matrix. Standards were incubated on ice for 30 min with vortexing for 5 s. A 4-fold standard dilution of the series and blank were prepared. Samples were vortexed for 5 s between liquid transfers. The 10x single beads were vortexed for 30 s and diluted to 1× in the Bio-Plex Assay Buffer. They were protected from light. The assays were run following the manufacturer’s instructions (Bio-Plex Pro Mouse Cytokine Grp, Catalog NO. #M60009RDPD, Bio-Rad Laboratories, Inc., U.S.A.). The assay measurements were conducted on the Bio-Plex MAGPIX System plate-reader (Bio-Rad Laboratories, Inc.). Finally, the C-C motif chemokine ligand 2 (CCL2), interleukin-6 (IL-6), and tumor necrosis factor α (TNFα) concentration were calculated by the standard curve.

### SDS-PAGE and Western blot

The methods for protein extraction from RAW264.7 cells, electrophoresis, and subsequent Western blotting are as described. The cells were lysed in RIPA buffer (high) (Cat No. #R0010, Beijing Solarbio Science & Technology Co., Ltd, China) containing halt protease and phosphatase inhibitor single-use cocktail for 30 min on ice. The lysates were centrifuged at 12,000 rpm for 30 min at 4°C. Each sample protein was separated on TGX Stain-Free FastCast Acrylamide Kit, 10% (Cat No. #1610183, Batch No. 64278801, Bio-Rad Laboratories, Inc., www.bio-rad.com, U.S.A.) and immunoblotting with antibodies (Table 1) followed by HRP-conjugated secondary antibody. Visualized bands were analysed on ChemiDocTMMP imaging system (Model No. Universal Hood III, Serial No. 731BR02915, Bio-Rad Laboratories, Inc., www.bio-rad.com, U.S.A.) using ImageLab™ software. Quantification was performed with Image Pro Plus software.

**Table 1:** Antibody information of the anti-NF-κB-p65, anti-CEBPB, anti-PPARG and anti-β actin in RAW264.7 cells

	Cat.No	Lot.No	Manufacturer	Specifications	Concentration
NF-κB p65	ab16502	GR165665-15	Abcam	100 μg	1 mg/ml
CEBPB	ab53138	GR3201578-6	Abcam	100 μg	1 mg/ml
PPARG	ab45036	GR3192046-8	Abcam	100 μl	1 mg/ml
β-Actin	TDY051F	/	TDY bio	100 μl	1 mg/ml

### Real-time quantitative polymerase chain reaction

NF-κB-p65, CEBPB, and PPARG mRNA levels were quantified by Real Time Quantitative PCR (RT-qPCR). According to the manufacturer’s instructions from Direct-zol™ RNA MiniPrep (Cat No. R2052, www.zymoresearch.com, U.S.A.), total RNA samples were extracted from the frozen mice liver tissues using TRIzol Reagent (Ref No. 15596026, Ambion by Life Technologies, U.S.A.). Total RNA was measured by NANODROP 2000 Spectrophotometer (ND2000, Thermo Scientific, Gene Company Limited). cDNA synthesis was carried out with the High Capacity cDNA Reverse Transcription Kit (Ref No. 4368814, applied biosystems by Thermo Fisher Scientific, Thermo Fisher Scientific Baltics UAB, Lithuania). Real-time qPCR was performed with the ABI 7900 system (applied biosystems, U.S.A.) using an Power SYBR Green PCR Master Mix (Ref No. 4367659, applied biosystems by Thermo Fisher Scientific, Life Technologies LTD, U.K.). The primers were synthesized by Thermo Fisher and the sequences were listed in Table 2. The thermal conditions of PCR were as follows: 1 cycle of 95°C for 10 min, 45 cycles of 95°C for 15 s, 55–60°C for 20 s, and 72°C for 30 s. The relative expression level of each gene was determined by the 2^−△△Ct^ method and normalized to the β-actin mRNA in each sample.

**Table 2:** Primer of the NF-κB-p65, CEBPB, PPARγ, and β-actin in RAW264.7 cells

Gene name	Full name	Region	Sequence
NF-κB p65 (mouse)	Nuclear factor- kappa B *p65*	Forward	5′-ATGGCAGACGATGATCCCTAC-3′
		Reverse	5′-CGGAATCGAAATCCCCTCTGTT-3′
CEBPB (mouse)	CCAAT enhancer binding proteins Beta	Forward	5′-GACAAGCTGAGCGACGAGTAC-3′
		Reverse	5′-TTGCGCATCTTGGCCTTGTC-3′
PPARG (mouse)	Peroxisome proliferators-activated receptors Gamma	Forward	5′-AAGAGCTGACCCAATGGTTGC-3′
		Reverse	5′-AGGTGGAGATGCAGGTTCTACTTTG-3′
β-Actin (mouse)	Beta cytoskeletal actin	Forward	5′-GTGACGTTGACATCCGTAAAGA-3′
		Reverse	5′-GCCGGACTCATCGTACTCC-3′

### Statistical analysis

Statistical analysis was performed with SPSS software 13.0 (IBM, Almon, NY, U.S.A.). For multiple comparisons, one-way analysis of variance (one-way ANOVA) was performed. All results were expressed as the mean ± SD. *P*-values <0.05, 0.01, or 0.001 were considered statistically significant. Graphs were plotted using GraphPad Prism Version 8.3 software.

## Results

### Chemical, physical, and toxic properties of AG

The chemical, physical and toxic properties of AG obtained from the PubChem database are shown in Tables 3 and 4, respectively. Table 3 summarizes some characteristics of AG important for its pharmacological functions, such as molecular weight and number of stereocenters. However, limited toxicological information on AG exists in the PubChem database; only one study of LD_50_ in mice has been reported (Table 4).

**Table 3:** Chemical and physical properties of AG

Property name	Property value
Molecular weight	350.455 g/mol
XLogP3-AA	2.2
Hydrogen bond donor count	3
Hydrogen bond acceptor count	5
Rotatable bond count	3
Exact mass	350.209 g/mol
Monoisotopic mass	350.209 g/mol
Topological polar surface area	87 A⁁2
Heavy atom count	25
Formal charge	0
Complexity	597
Isotope atom count	0
Defined atom Stereocenter count	6
Undefined atom Stereocenter count	0
Defined bond Stereocenter count	1
Undefined bond Stereocenter count	0
Covalently bonded unit count	1
Compound is canonicalized	yes

**Table 4:** Toxic properties of AG

Organism	Test type	Route	Dose	Reference
mouse	LD_50_	intraperitoneal	11,460 mg/kg	(Handa and Sharma, 1990)

Behavioural changes were not observed upon oral administration of AG for 14 days, nor was any mortality observed. Therefore, the LD_50_ value of AG was concluded to be greater than 5000 mg/kg in mice. As a result, AG showed no apparent acute toxicity in mice.

### Putative targets of AG

**Figure 2:**
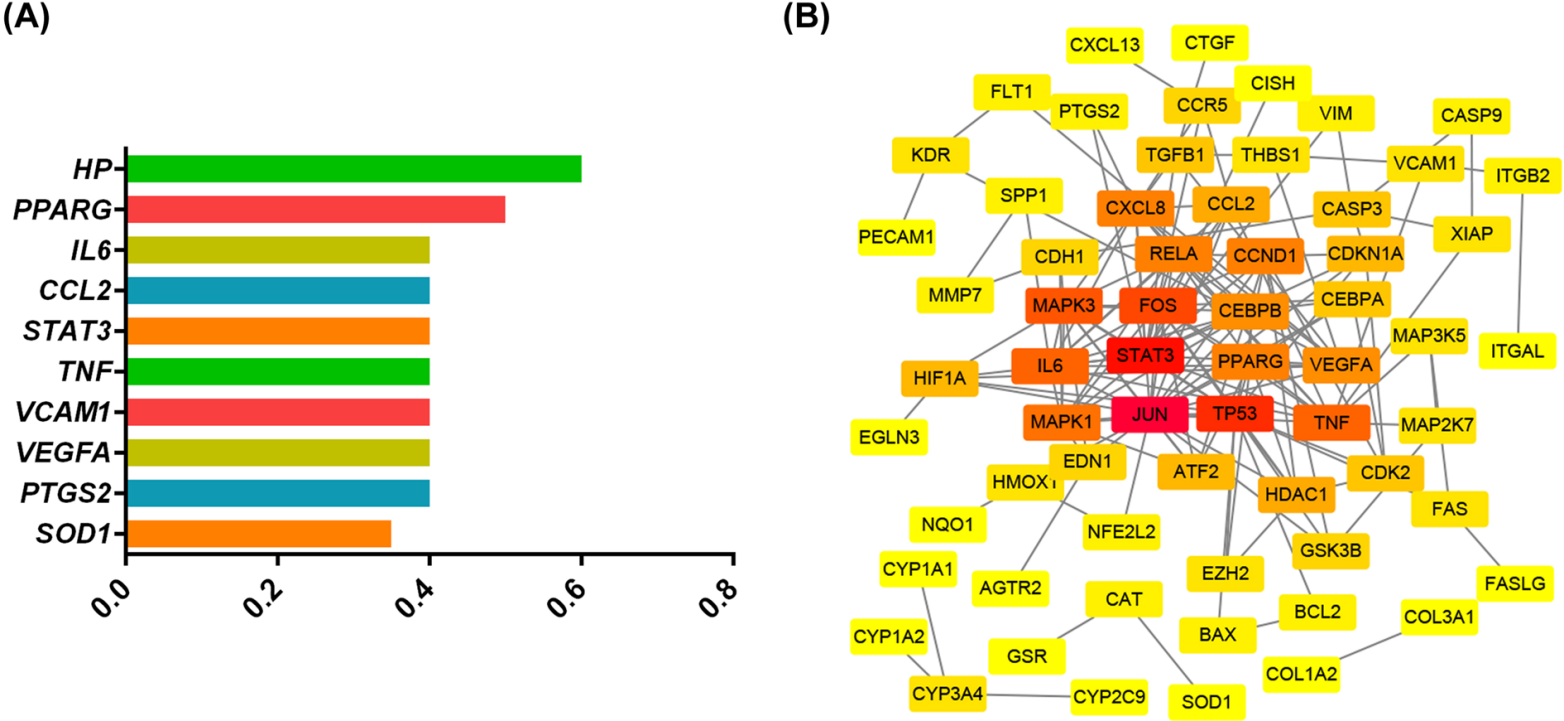
Importance of the target genes (**A**) Column diagram of genes according to GDA score. (**B**) PPI network. Color tones from yellow to red indicate lower to greater degree values.

After searching in different databases, as shown in Figure 1B, 146 genes associated with AG functions were collected from STITCH, STP, CTD, and BATMAN-TCM. Moreover, 2044 atherosclerosis-related genes were found in DisGeNet. Combining these findings, a column graph of highly related genes, according to the gene-disease association (GDA) score in DisGeNet, was generated as shown in Figure 2A. Eighty-one genes regulated by AG were associated with the development of atherosclerosis. The details of the 81 genes are summarized in Table 5.

**Table 5:** Information of target genes of AG on atherosclerosis

Num.	UniProt ID	Gene symbol	Gene name	Source
1	P62736	ACTA2	Actin alpha 2, smooth muscle	CTD
2	P18089	ADRA2B	Adrenoceptor alpha 2B	Stitch
3	P50052	AGTR2	Angiotensin II receptor type 2	Stitch
4	P15336	ATF2	Activating transcription factor 2	CTD
5	Q07812	BAX	BCL2 associated X, apoptosis regulator	CTD
6	P10415	BCL2	BCL2 apoptosis regulator	CTD
7	P42574	CASP3	Caspase 3	CTD
8	P55211	CASP9	Caspase 9	CTD
9	P04040	cat	Catalase	CTD
10	P13500	CCL2	C-C motif chemokine ligand 2	CTD
11	P29279	CCN2	Cellular communication network factor 2	CTD
12	P24385	CCND1	Cyclin D1	CTD
13	P51681	CCR5	C-C motif chemokine receptor 5 (gene/pseudogene)	Stitch
14	P51686	CCR9	C-C motif chemokine receptor 9	Stitch
15	P12830	CDH1	Cadherin 1	CTD
16	P24941	CDK2	Cyclin dependent kinase 2	CTD
17	P38936	CDKN1A	Cyclin dependent kinase inhibitor 1A	CTD
18	P49715	CEBPA	CCAAT enhancer binding protein alpha	CTD
19	P17676	CEBPB	CCAAT enhancer binding protein beta	CTD
20	Q6UWW8	CES3	Carboxylesterase 3	CTD
21	Q9NSE2	CISH	Cytokine inducible SH2 containing protein	CTD
22	Q99439	CNN2	Calponin 2	CTD
23	P08123	COL1A2	Collagen type I alpha 2 chain	CTD
24	P02461	COL3A1	Collagen type III alpha 1 chain	CTD
25	O43927	CXCL13	C-X-C motif chemokine ligand 13	CTD
26	P10145	CXCL8	C-X-C motif chemokine ligand 8	CTD
27	P04798	CYP1A1	Cytochrome P450 family 1 subfamily A member 1	CTD
28	P05177	CYP1A2	Cytochrome P450 family 1 subfamily A member 2	CTD
29	P11712	CYP2C9	Cytochrome P450 family 2 subfamily C member 9	CTD
30	P08684	CYP3A4	Cytochrome P450 family 3 subfamily A member 4	CTD
31	P05305	EDN1	Endothelin 1	CTD
32	Q9H6Z9	EGLN3	egl-9 family hypoxia inducible factor 3	CTD
33	Q15910	EZH2	Enhancer of zeste 2 polycomb repressive complex 2 subunit	CTD
34	P25445	FAS	Fas cell surface death receptor	CTD
35	P48023	FASLG	Fas ligand	CTD
36	P17948	FLT1	fms-related receptor tyrosine kinase 1	CTD
37	P01100	FOS	Fos proto-oncogene, AP-1 transcription factor subunit	CTD
38	P48507	GCLM	Glutamate-cysteine ligase modifier subunit	CTD
39	P49841	GSK3B	Glycogen synthase kinase 3 beta	CTD
40	P00390	GSR	Glutathione-disulfide reductase	CTD
41	P28161	GSTM2	Glutathione S-transferase mu 2	CTD
42	Q13547	HDAC1	Histone deacetylase 1	CTD
43	Q16665	HIF1A	Hypoxia inducible factor 1 subunit alpha	CTD
44	P04035	HMGCR	3-Hydroxy-3-methylglutaryl-CoA reductase	BATMAN
45	P09601	HMOX1	Heme oxygenase 1	CTD
46	/	HOTAIR	HOX transcript antisense RNA	CTD
47	P00738	HP	Haptoglobin	CTD
48	P05231	IL6	Interleukin 6	CTD
49	P20701	ITGAL	Integrin subunit alpha L	batman
50	P05107	ITGB2	Integrin subunit beta 2	batman
51	P05412	JUN	jun proto-oncogene, AP-1 transcription factor subunit	CTD
52	P35968	KDR	Kinase insert domain receptor	CTD
53	O14733	MAP2K7	Mitogen-activated protein kinase kinase 7	CTD
54	Q99683	MAP3K5	Mitogen-activated protein kinase kinase kinase 5	CTD
55	P28482	MAPK1	Mitogen-activated protein kinase 1	CTD
56	P27361	MAPK3	Mitogen-activated protein kinase 3	CTD
57	P09237	MMP7	Matrix metallopeptidase 7	CTD
58	Q16236	NFE2L2	Nuclear factor, erythroid 2 like 2	CTD
59	Q9NPG2	NGB	Neuroglobin	CTD
60	P15559	NQO1	NAD(P)H quinone dehydrogenase 1	CTD
61	P16284	PECAM1	Platelet and endothelial cell adhesion molecule 1	CTD
62	P37231	PPARG	Peroxisome proliferator activated receptor gamma	CTD
63	P17252	PRKCA	Protein kinase C alpha	STP
64	P05771	PRKCB	Protein kinase C beta	STP
65	P23219	PTGS1	Prostaglandin-endoperoxide synthase 1	STP
66	P35354	PTGS2	Prostaglandin-endoperoxide synthase 2	STP
67	Q04206	RELA	RELA proto-oncogene, NF-κB subunit	STP
68	P00441	SOD1	Superoxide dismutase 1	CTD
69	P10451	SPP1	Secreted phosphoprotein 1	CTD
70	Q13501	SQSTM1	Sequestosome 1	CTD
71	P40763	STAT3	Signal transducer and activator of transcription 3	CTD
72	Q687X5	STEAP4	STEAP4 metalloreductase	CTD
73	P48775	TDO2	Tryptophan 2,3-dioxygenase	CTD
74	P01137	TGFB1	Transforming growth factor beta 1	CTD
75	P07996	THBS1	Thrombospondin 1	CTD
76	P01375	TNF	Tumor necrosis factor	CTD
77	P04637	TP53	Tumor protein p53	CTD
78	P19320	VCAM1	Vascular cell adhesion molecule 1	CTD
79	P15692	VEGFA	Vascular endothelial growth factor A	CTD
80	P08670	vim	Vimentin	CTD
81	P98170	XIAP	X-linked inhibitor of apoptosis	CTD

### Analysis of protein–protein interaction (PPI)

The PPI network was established to better understand protein–protein interactions (Figure 2B). In this figure, disconnected nodes were not shown, and the confidence score was set to 0.95. The final network embodied 64 nodes and 156 edges; the degree were used to evaluate the importance of proteins in this network.

### GO enrichment

**Figure 3:**
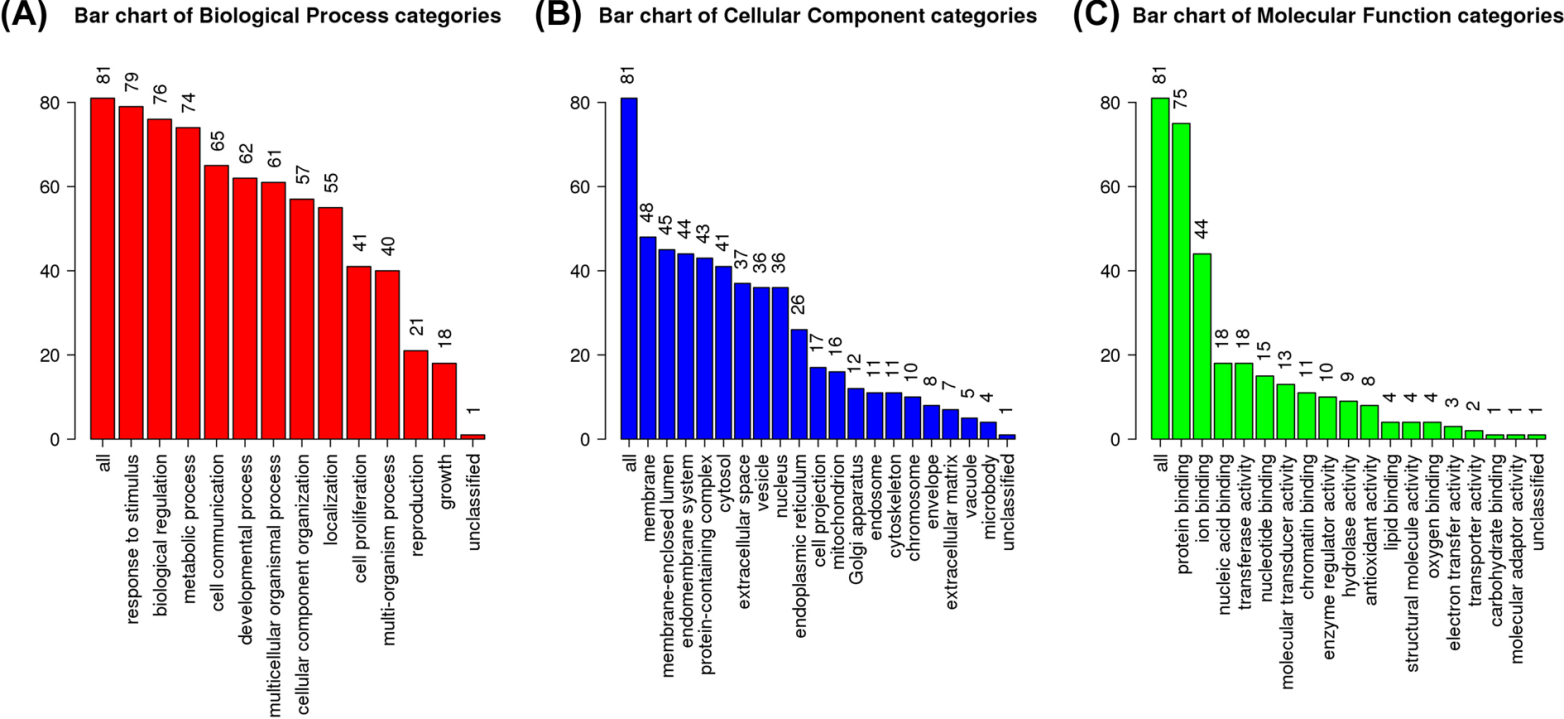
Results of GO analysis (**A**) Bar chart of Biological Process categories. (**B**) Bar chart of Cellular Component categories. (**C**) Bar chart of Molecular Function categories.

To further analyse the functions of the 81 target genes, GO enrichment was performed at WebGestalt (Figure 3). In this platform, the functions are divided into three levels: biological process, cellular component, and molecular functions. The top five genes were attributed to response to stimulus (79/81), biological regulation (76/81), protein binding (75/81), metabolic process (74/81), and cell communication (65/81).

### KEGG enrichment

**Figure 4:**
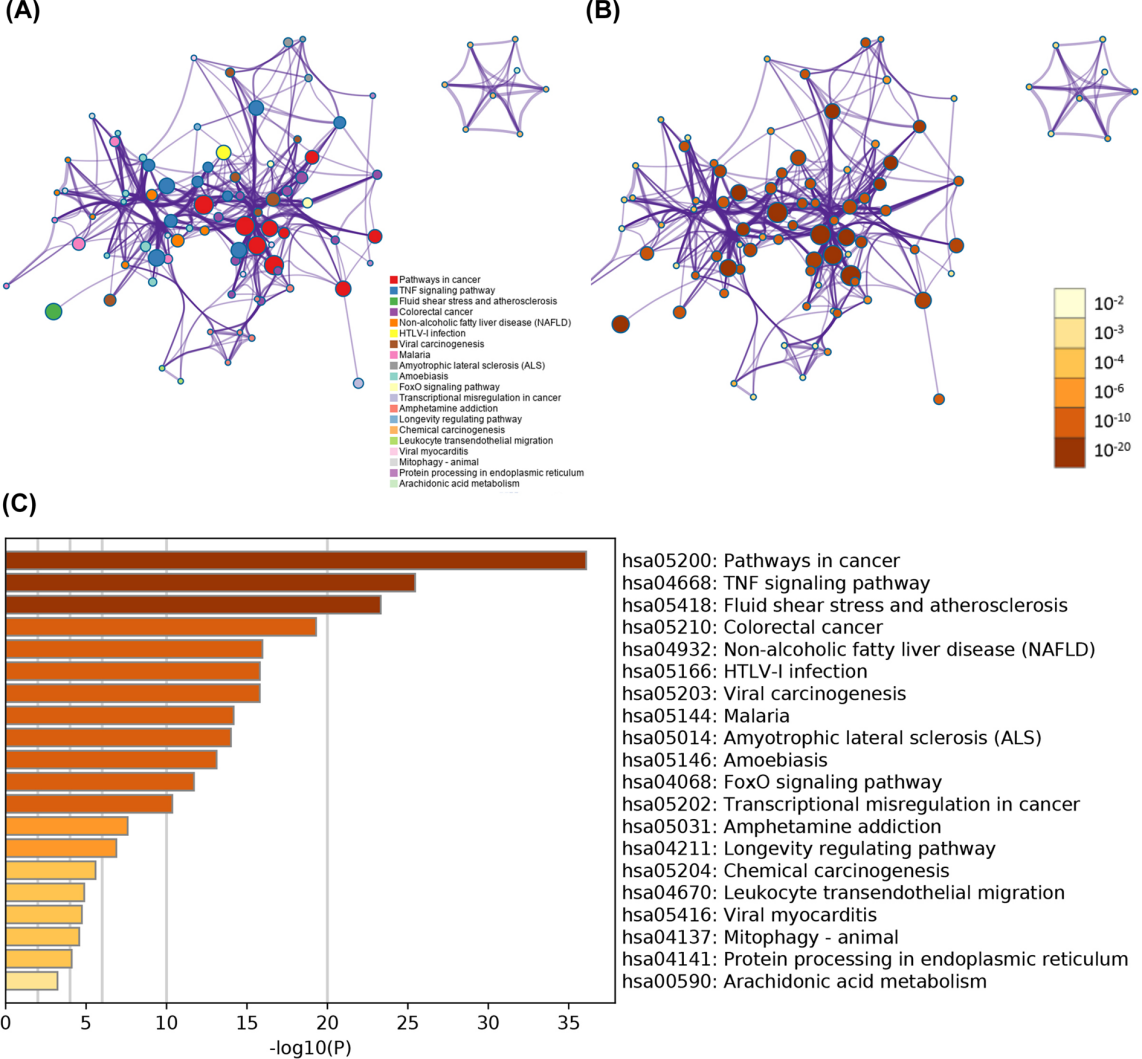
Results of KEGG pathway enrichment (**A**) Relationship between pathways (color by cluster). (**B**) Relationship between pathways (color by *P* value). (**C**) Heat map of pathways by rank.

A network figure was mapped using the Metascape platform to predict pathways involving the 81 target genes. As shown in Figure 4, the top five pathways affected by AG were pathways involved in cancer (hsa05200), TNF signaling pathway (hsa04668), fluid shear stress, atherosclerosis (hsa05418), colorectal cancer (hsa05210), and non-alcoholic fatty liver disease (NAFLD) (hsa04923). The interactions between the pathways and the heat map, based on rank, are also shown in Figure 4A–C.

### Network analysis

**Figure 5:**
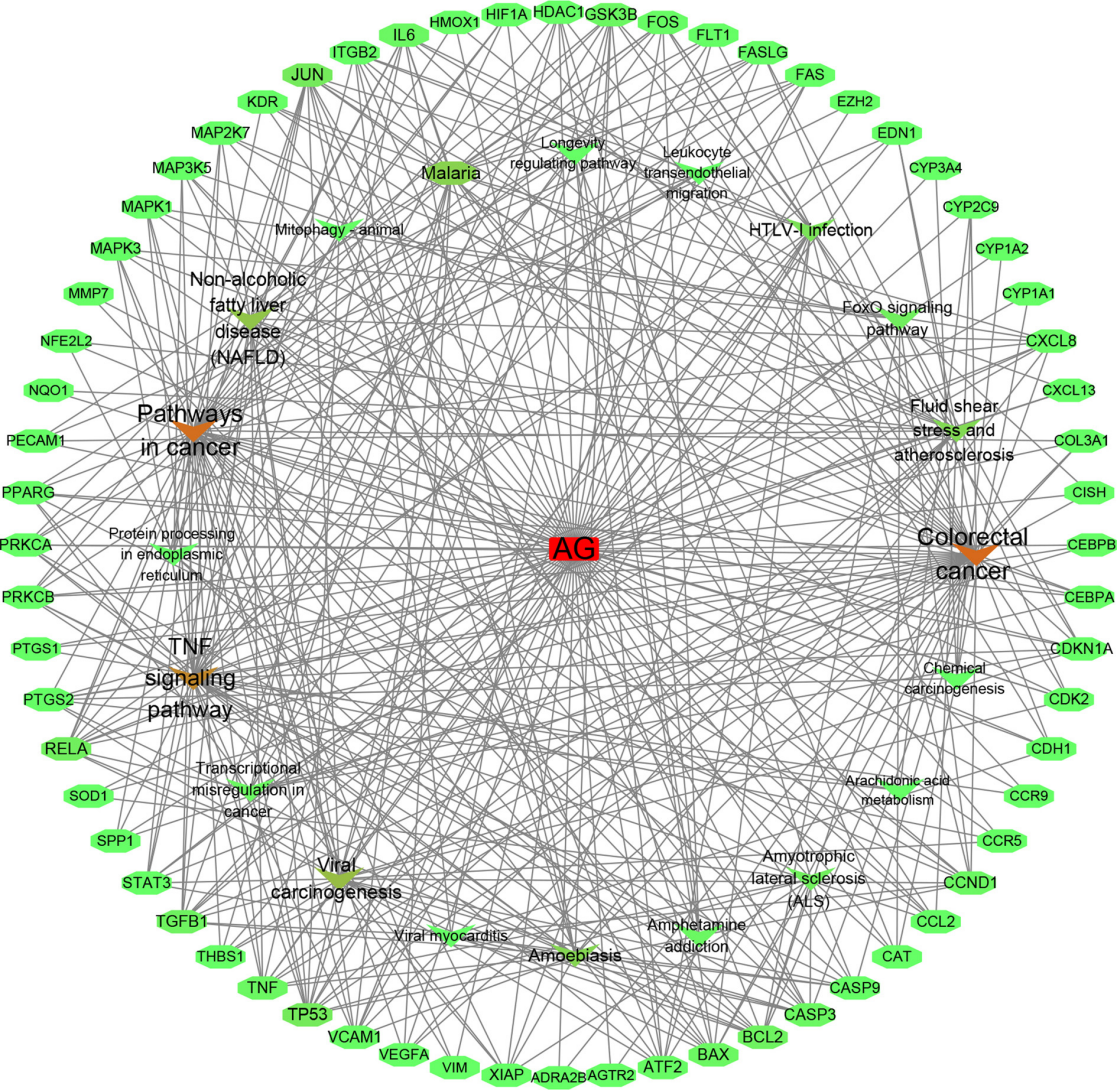
Compound-target-pathway network Rectangle nodes represent AG, ellipse nodes represent targets, arrow nodes represent pathways. The color from green to red depends on the degree value.

The three-layer network was constructed and analysed by Cytoscape 3.6.0, as shown in Figure 5. This network contains 85 nodes and 390 edges and shows the relationship between AG, target genes and pathways.

### Effect of Oil Red staining in RAW264.7 cells

**Figure 6:**
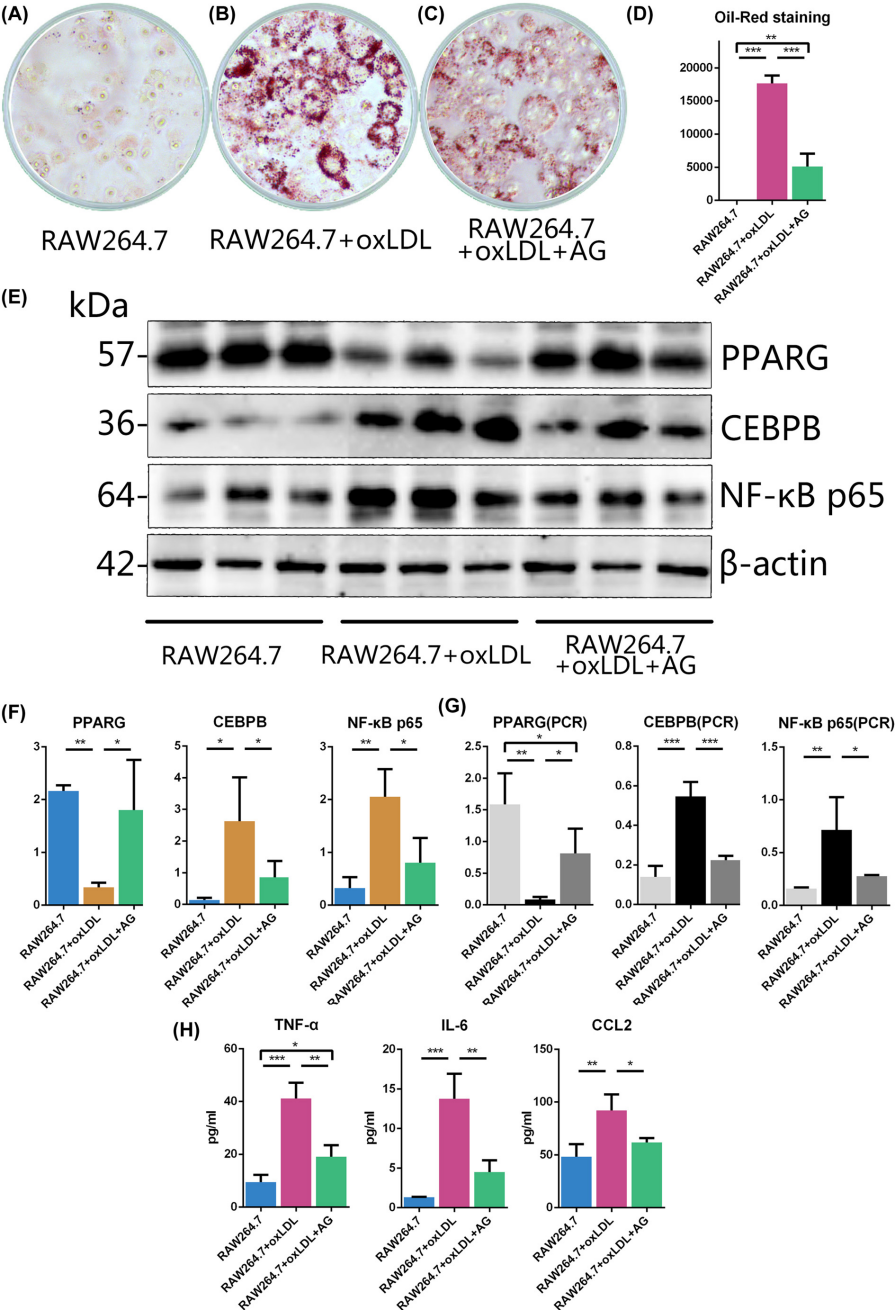
Results of the experimental verification (**A**) Untreated RAW264.7 cells after Oil Red O staining. (**B**) oxLDL treated RAW264.7 cells after Oil Red O staining. (**C**) oxLDL+AG treated RAW264.7 cells after Oil Red O staining. (**D**) The red area of RAW264.7 cells after Oil Red O staining (%), (*N* = 3 per group). (**E**) Electrophoretic bands of PPARG, CEBPB, and NF-κB p65. (**F**) Levels of protein expression (*N* = 3 per group). (**G**) Levels of mRNA expression (*N* = 3 per group). (**H**) Levels of inflammatory cytokines. The data are expressed as the mean ± SD of the mean and analysed by one-way ANOVA: *P<0.05, **P<0.01, ***P<0.001 versus RAW264.7+oxLDL group.

Oil Red O staining was performed to identify the foam cell formation under different drug concentrations, as shown in Figure 6A–C. The red area decreased significantly after intervention by AG. This result shown in Figure 6D confirms ROI analysis finding (*P*<0.001, compared with the RAW264.7+oxLDL group).

### Effect of AG on mRNA and protein expression in RAW264.7 cells

To assess the mechanism of AG for promoting the RCT, the protein expression and transcript levels of related genes were measured. In the Western blotting experiment, a clear separation of electrophoretic bands was observed (Figure 6E). Compared with the RAW264.7 group, the expression of PPARG in the RAW264.7+oxLDL group was significantly down-regulated (*P*<0.01); however, CEBPB (*P*<0.05) and NF-κB-p65 (*P*<0.01) were up-regulated. AG reversed the trend, as shown in Figure 6F (*P*<0.05). In the RT-qPCR experiment, the mRNA of PPARG in the RAW264.7+oxLDL group is significantly down-regulated (*P*<0.01) compared with that in the RAW264.7 group. In contrast, CEBPB (*P*<0.001) and NF-κB-p65 (*P*<0.01) are up-regulated. AG reversed the trend, as shown in Figure 6G (PPARG: *P*<0.05, CEBPB: *P*<0.001, NF-κB-p65: *P*<0.05). Besides, the RAW264.7+oxLDL+AG group also showed significantly lower PPARG mRNA than the RAW264.7 group (*P*<0.05).

### Effect of AG on inflammatory cytokines

The levels of cytokines are shown in Figure 6h. CCL2, IL-6, and TNF-α in the RAW264.7+oxLDL group were significantly higher than those in the RAW264.7 group (CCL2: *P*<0.01, IL6 and TNF-α: *P*<0.001). Additionally, AG inhibited the increase in CCL2, IL-6 and TNF-α in RAW264.7+oxLDL group(CCL2: *P*<0.05, IL6 and TNF-α: *P*<0.01). Besides, the TNF-α levels in the RAW264.7+oxLDL+AG group were also significantly higher than those in the RAW264.7 group (*P*<0.05).

## Discussion

Atherosclerosis is a crucial underlying pathology of diseases including coronary heart disease, myocardial infarction, stroke, and peripheral artery disease [38]. The aetiology of atherosclerosis is intrinsically linked to cholesterol accumulation at the arterial wall and is caused by an imbalance between deposition and removal [39]. Macrophage accumulation and cholesterol-rich plaques, characteristic of atherosclerosis, can be simulated in oxLDL-induced RAW264.7 cells. Currently, drugs with lipid-lowering and anti-atherosclerotic effects are commonly used in the clinic; however, their use has been accompanied by an increase in liver and kidney damage and other adverse effects [40]. Therefore, there is a great need for developing safe and effective new drugs for patients who cannot tolerate these adverse effects. Previous studies have shown that natural drugs and their derived compounds play an essential role in regulating lipid metabolism and atherosclerosis [41,42]. AG, isolated from *A. paniculata*, could ameliorate lipid metabolism disorder and atherosclerosis. It is known to exert multifaceted antiarteriosclerotic effects, such as anti-inflammatory, inhibiting the formation of foam cells, antiplatelet and protecting the endothelium [43–46]. The activation of eNOS-NO / cGMP [45], inhibition of PI3 kinase/Akt-p38 MAPK pathway [46] and NF-κB pathway [43] are reportedly involved. However, the underlying molecular mechanism has not been fully understood. Network pharmacology, a powerful tool to identify alternative targets for herbal medicines, has advantages in facilitating the development of new drugs and assessing their safety [47,48]. In the present study, a total of 146 predicted target genes in AG were obtained. Among these targets, 81 genes were associated with atherosclerosis. In addition, we demonstrate a synergistic treatment strategy for AG featuring multi-target and multi-pathways by applying various methods, including PPI, GO analysis, and Compound-Target-Pathway network. Finally, we performed experiments in RAW264.7 cells to validate part of the mechanism.

First, Lipinski’s rule of five is generally referred to as a guideline for drug optimization. It provides relevant scope to help assess the properties of drugs [49,50]. As shown in Table 1, the pharmacokinetic properties of AG meet these requirements, suggesting that it has suitable medicinal properties for the development of new commercially available drugs with high efficacies. An optimized AG-loaded nanoemulsion was developed in recent research to improve AG oral bioavailability. The pharmacokinetic results indicate that the AG-loaded nanoemulsion significantly enhanced the absorption of AG in comparison with the AG suspension with a relative bioavailability of 594.3% [51]. Moreover, solid lipid nanoparticles can increase the delivery of AG into the brain, which significantly enhances the rate of AG crossing the blood–brain barrier compared with that of free AG [52], providing strong support for the expansion of AG’s efficacy.

Second, pathogenic mechanisms and interactions of potential targets were also elucidated in our study. As shown in Figure 2A, targets with a high GDA score are closely related to chronic inflammation in atherosclerosis. Figure 2B analyses the interaction according to the degree. The potential antiatherosclerotic properties of AG are probably mediated by two different types of proteins. One type is closely related to tumor proliferation, possibly through JUN, STAT3, TP53, FOS, MAPK3, MAPK1, PPARG, and CCND1. The other types of proteins, like IL6, TNF, RELA, CXCL8, and CEBPB, are associated with the regulation of inflammation. A subset of proteins performs the two distinct functions simultaneously. The regulation of the above targets by AG occurs in numerous diseases, especially cancer. TP53 genes that correlate significantly with STAT3 may be especially critical nodes in human cancers [53,54]. In human cervical and colorectal cancer cells, TP53 and STAT3 are simultaneously regulated by miR-214 [55]. JUN and FOS are the subunit proteins of the AP-1 transcription factor. AG suppresses angiogenesis in the tumour microenvironment in HCT116 colorectal cancer cells partly because of the inhibition of AP-1 and MAPKs expression [56]. AG also inhibits cell-cycle progression in human colorectal carcinoma lovo cells with a marked decrease in the protein expression of CCND1. Although cancer increases the risk of many other diseases, the mechanism of tumour progression is too complex to interpret as atherosclerosis. Notably, PPARG is a ligand-activated nuclear transcription factor that has a central role in controlling lipid metabolism [57]. PPARG signaling has also been implicated in the control of atherosclerosis [57]. Research about the effects of AG on PPARG activation focusses chiefly on lipid metabolism and the inflammatory response, which may be more closely related to atherosclerosis.

Third, the GO project and KEGG pathway enrichment analysis revealed the correlation of 81 target genes with their response to stimulus, biological regulation, protein binding, metabolic process, and cell communication. They regulate lipid transport [58], ameliorate inflammation [59], attenuate oxidative stress, and promote anti-tumour effect [60], which are intersecting, interacting, and synergetic as described in the literature.

The compound-target-pathway network shown in Figure 5 further indicated that AG plays different roles in multiple targets or pathways.

Fourth, AG-treated foam cell formation seemed to be significantly decreased, as demonstrated by Oil Red O staining. Thus, AG may be involved in macrophage cholesterol efflux, which is the first step of RCT. AG inhibits inflammation by interacting with NF-κB, the factor which regulates the transcription of pro-inflammatory cytokine genes, such as those coding for TNF-α, IL-6, and CCL2 [61]. Similar observations have been described for CEBPB interaction with NF-κB. Activation of CEBPB is induced by up-regulation of NF-κB [62]. Notably, the down-regulation of CEBPB has been reported to induce PPARG in vascular protection [63]. CEBPB and PPARG modulate macrophage function, including M2 macrophage polarization [64]. They are also similarly involved in lipid metabolism [65]. Activation of both could contribute, at least in part, to the amelioration of lipid homeostasis and atherosclerosis [66]. The variation in the expression of the above three genes has established a role for AG in the regulation of lipid metabolism and inflammation in atherosclerosis.

In conclusion, we successfully predict the potential targets of AG for application in atherosclerosis and help illustrate the mechanism of action on a systematic level. This study not only provides new insights into the chemical basis and pharmacology of AG but also demonstrates a feasible method for evaluating potential molecular drugs from herbal medicine. Our results show that AG may regulate NF-κB/CEBPB/PPARG signalling to play a therapeutic role in atherosclerosis.

## Data Availability

Some or all data, models or used during the study are available from the corresponding author by request. Direct requests for these materials may be made to the provider as indicated.

## Abbreviations

AG: andrographolide
AS: atherosclerosis
ASCVD: atherosclerotic cardiovascular disease
BATMAN-TCM: A bioinformatics analysis tool for molecular mechANism of traditional Chinese medicine
CAD: coronary artery disease
CTD: Comparative Toxicogenomics Database
GDA: gene-disease association
GO: Gene ontology
IL-6: interleukin-6
KEGG: Kyoto encyclopedia of genes and genomes
NAFLD: nonalcoholic fatty liver disease
OMIM: Online Mendelian Inheritance in Man
one-way ANOVA: one-way analysis of variance
OSA: overrepresentation enrichment analysis
oxLDL: oxidized low-density lipoprotein
PPI: protein–protein interaction
RCT: reverse cholesterol transport
RT-qPCR: real-time quantitative PCR
STP: Swiss target predition
TCM: traditional Chinese medicine
TNF: tumor necrosis factor
WebGestalt: WEB-based GEne SeT AnaLysis toolkit
